# Enhancing Mechanical Properties of Graft-Type Nanocomposites Using Organically Modified SiO_2_ and Polypropylene Containing Reactive Methoxy Groups

**DOI:** 10.3390/polym14030563

**Published:** 2022-01-30

**Authors:** Dongzhi Zhu, Eiji Kurahashi, Hui You, Toru Wada, Patchanee Chammingkwan, Toshiaki Taniike

**Affiliations:** 1 Graduate School of Advanced Science and Technology, Japan Advanced Institute of Science and Technology, 1-1 Asahidai, Nomi 923-1292, Ishikawa, Japan; s1920417@jaist.ac.jp (D.Z.); s1920035@jaist.ac.jp (H.Y.); toruwada@jaist.ac.jp (T.W.); 2 Kojima Industries Corporation, 3-30 Shimoichiba-cho, Toyota 471-8588, Aichi, Japan; eiji-kurahashi@kojima-tns.co.jp

**Keywords:** PP nanocomposites, in situ grafting, reactive matrix, surface modification

## Abstract

In situ grafting of a reactive matrix and nanofillers is a promising strategy to fabricate graft-type polypropylene (PP)-based nanocomposites, where the grafting efficiency is affected by the initial dispersion of nanofillers in the matrix. In this work, influences of surface organic modification of nanofillers were investigated on properties of PP/SiO_2_ nanocomposites using poly(propylene-co-octenyltrimethoxysilane) as a reactive matrix. The surface modification of SiO_2_, especially with longer alkyl chains, led to improved dispersion of nanoparticles, thus promoting the grafting reaction and mechanical properties. The combination of in situ grafting and surface modification of nanofillers provided several benefits, most notably in balancing the strength and the toughness, which could not be achieved by the grafting alone.

## 1. Introduction

Polymer nanocomposites, consisting of at least one phase having dimensions smaller than 100 nm [1], have attracted great interest owing to their potential applications in different industrial sectors. Significant increases in the particle number density and interfacial area due to the small size enable nanocomposites to achieve desired properties at a much smaller loading of fillers compared to conventional microcomposites [2]. Meanwhile, strong interparticle attraction in comparison to weak interfacial interaction with polymer matrices constitutes one of the main impediments in the development of high-performance nanocomposites.

Polypropylene (PP), one of the most abundant thermoplastics, has been employed for fabricating polymer nanocomposites using a wide variety of nanofillers [3,4,5,6,7,8,9,10,11,12,13,14,15,16]. The hydrophobicity and chemical innerness of PP make uniform dispersion of nanoparticles challenging, for which various strategies have been reported. These include the use of a compatibilizer such as maleic anhydride-grafted PP [17,18,19,20,21,22,23,24,25], surface modification by organic compounds [26,27,28], polymer grafting [29,30,31,32,33], propylene polymerization in presence of nanofillers [34], in situ formation of nanofillers [35,36,37], and so on. Among these, the polymer grafting, especially using PP as the graft chain, is the most promising [38]. Grafted PP chains not only improve the compatibility between nanofillers and the PP matrix, they also co-crystallize with the matrix to strengthen interfacial interaction [38]. It has been reported that various functional groups have been introduced to PP chains as a reactive site for grafting reaction [38,39,40,41,42,43]. For example, we exploited terminally hydroxylated PP (PP-*t*-OH) with different chain lengths, which was synthesized through controlled chain transfer reaction during metallocene-catalyzed propylene polymerization, followed by hydroxylation. PP-*t*-OH was grafted onto silica nanoparticles (SiO_2_) and subsequently used for melt mixing with a PP matrix [38]. The resultant nanocomposites exhibited excellent dispersion of nanoparticles and significant reinforcement arisen from co-crystallization-based physical crosslinkage. Nonetheless, elaboration in the synthesis of PP-*t*-OH as well as ex situ grafting makes the process less practical. In a more recent publication, efficient synthesis of reactive PP with less than one functional group per chain by virtue of catalyzed copolymerization between propylene and 7-octenyltrimethoxysilane (OTMS) was reported [39]. During melt mixing, PP-OTMS undergoes in situ grafting onto SiO_2_ through the reaction between methoxy groups at a side chain and silanol groups on SiO_2_ surfaces, leading to improved dispersion and reinforcement, similar to ex situ grafting but more efficiently. In addition, interchain reaction among methoxy groups creates a crosslink network, which also co-contributes to the improvement in mechanical properties.

Despite the effective reinforcing of PP-OTMS/SiO_2_ nanocomposites, due to particle-particle interactions and the high surface energy of SiO_2_, uniform dispersion of neat SiO_2_ in the early stages of melt mixing remains a challenge [26,44]. The shear forces during melt mixing were unable to break down this agglomeration of SiO_2_ in the matrix. Surface modification of SiO_2_ with inert functional groups, such as alkyl chains, is the most convenient method for weakening connections between adjacent nanoparticles and decreasing moisture absorption by lowering the surface energy [44]. As the grafting reaction and interchain reaction are competing reactions that consume the reactive functional group [39], good control of SiO_2_ dispersion in the early stage would provide more opportunities to control the grafting reaction and to maximize the resultant properties.

Here, we report a method to further improve the physical properties of graft-type nanocomposites using PP-OTMS as reactive matrix. Specifically, organically modified SiO_2_ nanoparticles were used in combination with PP-OTMS. The organic modification improved the dispersion of the nanoparticles in the early stage of melt mixing and consequently improved the grafting efficiency. This, together with the plasticizing effect of aliphatic chains, resulted in improved mechanical properties compared to the nanocomposites using unmodified nanoparticles. In particular, the improvement in toughness was quite remarkable.

## 2. Materials and Methods

### 2.1. Materials

PP (*M*_n_ = 6.3 × 10^4^, *M*_w_/*M*_n_ = 3.9, stereoregularity *(mmmm)* = 95 mol%) and PP-OTMS (*M*_n_ = 7.3 × 10^4^, *M*_w_/*M*_n_ = 3.7, *mmmm* = 98 mol%) were synthesized using a 5th-generation Ziegler-Natta catalyst according to our previous study [39]. *n*-Heptane was dried by N_2_ bubbling in the presence of molecular sieve 3A prior to use. Propylene of polymerization grade was donated by Japan Polypropylene Corporation (Tokyo, Japan) and used as received. Triethylaluminum (TEA, donated by Tosoh Finechem Corporation, Yamaguchi, Japan) was used as a dilution in heptane. 7-octenyltrimethoxysilane (OTMS, purity > 90%, Tokyo Chemical Industry Co., Ltd., Toyko, Japan) was used as a comonomer without further purification. Based on a gas chromatography-mass spectrometry analysis, the impurities in OTMS were OTMS isomers with different double bond positions. These impurities are similarly or less poisonous to the catalyst as compared to OTMS. Therefore, they are assumed to have insignificant effects on the catalytic properties. SiO_2_ nanoparticles (average diameter = 26 nm, specific surface area = 110 m^2^ g^−1^) were purchased from Kanto Chemical Co., Inc. (Tokyo, Japan) Octadecyl-3-(3,5-di-*tert*-butyl-4-hydroxyphenyl)propionate (AO-50, donated by ADEKA Corporation, Tokyo, Japan) was used as a stabilizer. Trichlorohexylsilane, trichlorododecylsilane, and trichlorohexadecylsilane were purchased from Tokyo Chemical Industry Co., Ltd. (Tokyo, Japan). According to the alkyl chain length, they are denoted as C6, C12, and C16, respectively. Toluene (Kanto Chemical Co., Inc., Tokyo, Japan) was dried and deoxygenated by N_2_ bubbling in the presence of molecular sieve 3A.

### 2.2. Synthesis of PP-OTMS

PP-OTMS was synthesized by copolymerization of propylene and OTMS using a MgCl_2_-supported Ziegler-Natta catalyst [39]. The polymerization was performed in a 1 L stainless steel reactor in a semi-batch mode. To the reactor blanked by N_2_, 500 mL of heptane as a solvent, 15 mmol of TEA as a cocatalyst, and 10 mmol of OTMS as a comonomer were introduced. The solvent was saturated with 0.5 MPa of propylene at 50 °C for 30 min. Followed by the introduction of 16 mmol of H_2_, catalyst powder (50 mg) was injected to initiate the polymerization. The polymerization was continued at 50 °C and 0.5 MPa for 60 min, followed by depressurization. The solvent was removed by decantation, and the resultant polymer powder was washed repetitively with ethanol and acetone under N_2_. Finally, the powder was purified by reprecipitation (xylene to acetone) and dried in vacuum at room temperature. PP homopolymer (HomoPP) was synthesized in the absence of OTMS under the same conditions, except 5.0 mmol of TEA used as a co-catalyst.

### 2.3. Surface Modification of SiO_2_

Surface modification of SiO_2_ was performed according to a previously reported method [45]. SiO_2_ (0.3 g) was added in 30 mL of toluene in a round-bottom flask, and sonicated for 30 min. A specified amount of a silane coupling agent (0.01, 0.1, or 1 mmol) was added under N_2_. The mixture was stirred at room temperature for 1 h. The obtained product was collected by centrifugation, repetitively washed with anhydrous ethanol, and dried in a vacuum oven at 80 °C for 24 h. The modified SiO_2_ samples are denoted as C*x-y*-SiO_2_, where *x* and *y* represent the alkyl chain length and the amount (mmol) of the silane coupling agents (per 0.3 g of SiO_2_), respectively. For example, C6-0.1-SiO_2_ stands for SiO_2_ modified with 0.1 mmol of trichlorohexylsilane.

### 2.4. Preparation of PP-OTMS/SiO_2_ Nanocomposites

PP-OTMS/SiO_2_ nanocomposites were prepared by the following procedure: First, PP-OTMS (3.7 g) was pre-impregnated with 1.0 wt% of AO-50 in 100 mL of acetone. After evaporation of the solvent under N_2_ flow, the polymer was dried at room temperature under vacuum for 12 h. The dried polymer was melt-mixed with 5.0 wt% of unmodified SiO_2_ or modified SiO_2_ using Micro Compounder MC5 (Xplore) at 185 °C and 100 rpm for 15 min under N_2_ atmosphere. The extrudate was hot-pressed into a 200 μm-thick film at 230 °C and 20 MPa for 5 min, followed by quenching at 100 °C for 5 min and then cooling at 0 °C for 3 min. For comparison, a nanocomposite (HomoPP/SiO_2_) was also prepared using HomoPP and unmodified SiO_2_ according to the same procedure.

### 2.5. Characterizations

Fourier transform infrared (FTIR) spectra were recorded on a Perkin Elmer Spectrum 100 spectrometer (PerkinElmer, Inc., Waltham, MA, USA) in the transmission mode between 4000 cm^−1^ and 450 cm^−1^ with a resolution of 4 cm^−1^. The scans of each FTIR experiment are 64. A sample was mixed with dried KBr and pressed into a disc for the measurement. Water contact angle (WCA) measurements were carried out on SiO_2_ samples using a contact angle goniometer (SImageAUTO 100, Excimer. Inc., Kanagawa, Japan). At room temperature, 10.0 μL of deionized water was dropped on a sample surface pressed on a glass slide. The WCA was determined by the tangent method. The weight loss of unmodified and modified SiO_2_ was measured by thermal gravimetric analysis (TGA, Thermo plus evo, Rigaku, Tokyo, Japan). A sample was heated from 25 °C to 600 °C at the heating rate of 10 °C min^−1^ under dry air flow. The weight loss below 200 °C corresponds to the evaporation of physisorped water and surface hydroxyl groups, while the weight loss in a range of 200–600 °C corresponds to thermal decomposition of organic groups for modified SiO_2_.

The methoxy (OMe) content in PP-OTMS before and after melt mixing was analyzed by ^1^H NMR (Bruker 400 MHz) operated at 120 °C with 1000 scans. Ca. 60 mg of a sample was dissolved in 0.2 mL of 1,1,2,2-tetrachloroethane-*d*_2_ (an internal lock and a reference) and 0.5 mL of 1,2,4-trichlorobenzene containing 0.006 wt% of 2,6-di-*tert*-butyl-4-methylphenol (anti-oxidant). The OMe content was calculated using Equation (1),

(1) $$\mathrm{OMe}\;\mathrm{content}\;(\mathrm{mol}\%)=\frac{H^{a}/3}{H^{c}}\;\times 100$$

where H^a^ and H^c^ are the peak areas for the methyl protons of the methoxy group and the methine protons of the polymer backbone, respectively. A typical NMR spectrum of PP-OTMS and peak assignment are provided in Figure S1.

The dispersion of SiO_2_ nanoparticles was observed on a transmission electron microscope (TEM, Hitachi H-7650, Hitachi High-Tech Corporation, Tokyo, Japan) operated at an acceleration voltage of 100 kV. A 100-nm-thick specimen was cut from a nanocomposite film using an ultramicrotome instrument (Leica ULTRACUTS FCS, Leica Microsystems GmbH, Wetzlar, Germany) equipped with a diamond knife. The dispersion of SiO_2_ in the matrix was quantitatively evaluated based on a dispersion parameter (*D*) defined in Equation (2),

(2) $$D=\frac{0.2}{\sqrt{2\pi }}\;\times \;\frac{\mu }{\sigma }$$

where *μ* and *σ* are the average size and its standard deviation of SiO_2_ domains (SiO_2_ particles or their aggregates) [46]. The analysis of TEM images was performed using ImageJ software, and covered at least 200 domains of SiO_2_, which corresponded to 3 TEM images taken at different regions and were sufficient to obtain stable *D* values.

Differential scanning calorimetry (DSC) measurements were performed on Mettler Toledo DSC 822 under N_2_ atmosphere. Ca. 8 mg of a sample was added in an aluminum pan, and heated to 230 °C at the heating rate of 20 °C min^−1^. The melting temperature (*T*_m_) and the crystallinity (*X*_c_) were determined from the melting endotherm. After holding 230 °C for 10 min, the sample was cooled down to 25 °C at the cooling rate of 20 °C min^−1^ for acquiring the crystallization temperature (*T*_c_), or to 144 °C at the rate of 50 °C min^−1^ for isothermal crystallization. In the latter case, the crystallization rate was calculated as the inverse of the half time of the crystallization (denoted as *t*_1/2_^−1^).

Tensile properties were measured using a tensile tester (Abecks Inc., Dat-100, Tokyo, Japan) at a crosshead speed of 1 mm min^−1^ at room temperature. Dumbbell-shaped specimens were die-cut from a 200-µm-thick film. The tensile properties were reported as an average from at least four measurements per sample.

## 3. Results and Discussion

**Figure 1:**
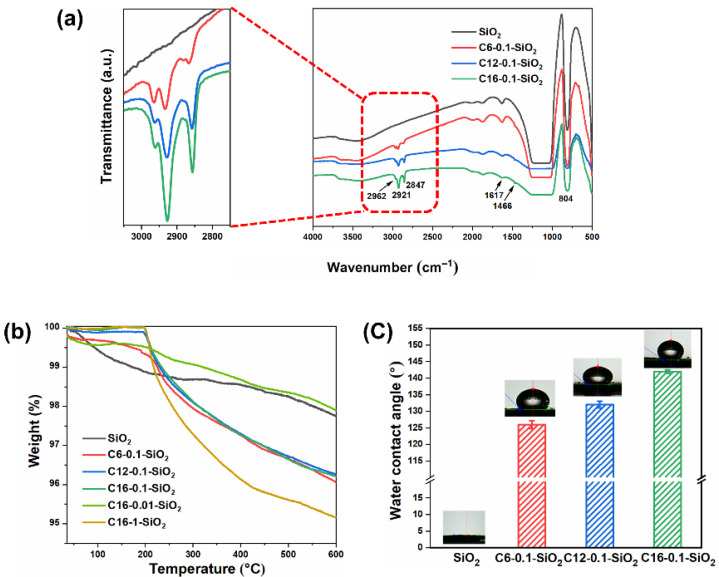
Characterization of unmodified and organically modified SiO_2_ nanoparticles: (**a**) FTIR spectra, (**b**) TG curves, and (**c**) water contact angles.

Successful organic modification of SiO_2_ was confirmed by FTIR. As shown in Figure 1a, unmodified SiO_2_ exhibited broad peaks at around 3500 cm^−1^ and 1617 cm^−1^, which are characteristics of surface hydroxyl groups and physisorbed water [47]. The most intense peak around 1100 cm^−1^ and the sharp peak at 804 cm^−1^ are respectively attributed to Si-OH/Si–O–Si stretching and Si–O–Si bending [48,49]. After surface modification, new peaks appeared at 2962 cm^−1^, 2921 cm^−1^, 2847 cm^−1^, and 1466 cm^−1^. These correspond to asymmetric stretching of −CH_3_, asymmetric stretching of −CH_2_, symmetric stretching of −CH_2_, and C−H bending [50]. It is also noted that the peak ratio between the asymmetric stretching of −CH_2_ and −CH_3_ changed in line with the CH_2_/CH_3_ ratio of the alkyl chain [47,48]. The morphology of the neat and surface modified SiO_2_ was conducted. It was found that the shape and size of the SiO_2_ before and after modification do not have changed (Figure S2).

TGA was implemented to determine the amount of silanes grafted on SiO_2_ surfaces. For unmodified SiO_2_, the weight loss gradually occurred upon heating to 600 °C, due to the vaporization of physisorbed water and the loss of surface hydroxyl groups [51]. For modified SiO_2_, a sharp weight loss was observed at around 200 °C, as shown in Figure 1b. According to literature, the weight loss of silane-modified SiO_2_ starts at around 200 °C via dissociative combustion of the alkyl chain, leaving the siloxy groups on the SiO_2_ surface [52]. The following equation was used to estimate the amount of grafted silanes from the weight loss in the range of 200–600 °C,

(3) $$m\%=\frac{1}{1+\frac{M_{\mathrm{Silane}}}{M_{{\mathrm{SiO}}_{2}}}\;\times \;\left(\frac{r_{a/s}}{\Delta W}\;-\;1\right)}\times 100$$

where *m*% is the molar percentage of silanes with respect to SiO_2_. *M*_silane_ and *M*_SiO_2__ are the molecular weights of silane coupling agents and SiO_2_, respectively. Δ*W* is the weight loss of modified SiO_2_ between 200–600 °C minus that of unmodified SiO_2_. *r*_a/s_ is the weight ratio of the alkyl chain in the correspondent silane coupling agent [52]. For example, Δ*W* is calculated as 3.27 wt% for C6-0.1-SiO_2_. The *r*_a/s_ and *M*_silane_ values for trichlorohexylsilane are 0.39 and 219.61 g mol^−1^, respectively. According to Equation (3), the molar percentage of the grafted silane is calculated as 1.57 mol%. Considering the surface area of SiO_2_, the silane grafting density can be further calculated as 1.43 nm^−2^. Table 1 summarizes the silane molar percentage and the grafting density when silane coupling agents having different alkyl chain lengths were employed. It can be seen that at the fixed mole of silane coupling agents (0.1 mmol), the grafting amount decreased with the increase in the alkyl chain length. It is considered that the adsorption and reaction of a molecule of a bulky silane coupling agent inhibits the approach of a new molecule to the surrounding silanols. By increasing the amount of the silane coupling agent (C16), the grafting density increased and reached 0.94 nm^−2^ at 1 mmol of the silane addition. It must be noted that the grafting density was sufficiently lower than the original OH density (ca. 4–5 nm^−2^) for all cases, i.e., the OH sites remained available for grafting to PP-OTMS.

Figure 1c depicts the results of WCA measurements for SiO_2_ samples. Without organic modification, the SiO_2_ surface was highly hydrophilic (WCA = 0°) due to the presence of surface hydroxyl groups. The modification with silane coupling agents significantly increased WCAs to 126–142°, indicating that the original hydrophilic surface changed to hydrophobic one due to the presence of aliphatic chains. The WCAs were found to be larger when the chain length increased from C6 to C12 regardless of a lower grafting density. These results pointed out that it is easier for a longer alkyl chain to shield the surface from water by forming a hydrophobic network [53].

**Figure 2:**
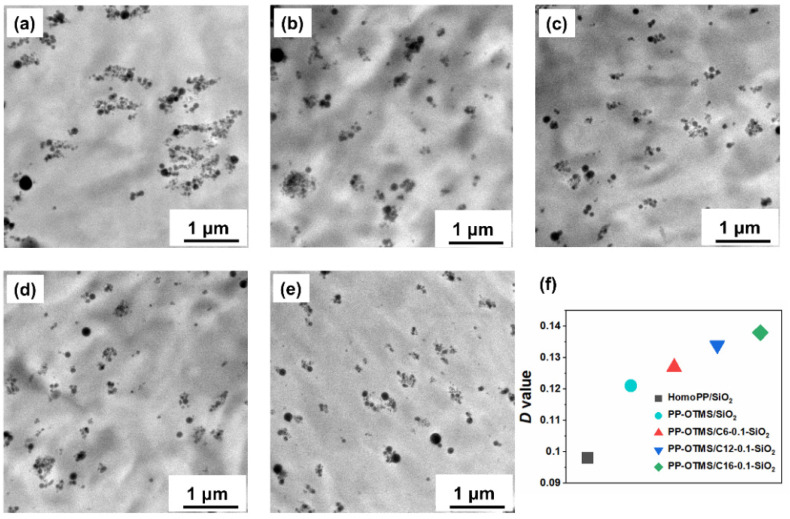
TEM images of nanocomposites: (**a**) HomoPP/SiO_2_, (**b**) PP-OTMS/SiO_2_, (**c**) PP-OTMS/C6-0.1-SiO_2_, (**d**) PP-OTMS/C12-0.1-SiO_2_, and (**e**) PP-OTMS/C16-0.1-SiO_2_. The SiO_2_ content was 5.0 wt% for all the samples. (**f**) The dispersion parameter (*D*) acquired from TEM images.

A series of nanocomposites were prepared by melt compounding 5.0 wt% of unmodified or modified SiO_2_. Figure 2 shows TEM images of the resultant nanocomposites and the dispersion parameter (*D*) calculated based on Equation (2). Generally, a higher *D* value indicates better dispersion [46]. As known from literature, SiO_2_ nanoparticles have poor compatibility with the PP matrix and easily form agglomerates due to strong particle-particle interactions (Figure 2a). The incorporation of a small amount of OTMS in PP chains helped to improve the dispersion as evidenced by the decrease in the size of SiO_2_ domains (Figure 2b). In our previous paper, the improved dispersion was ascribed to the presence of polar functional groups as well as the in situ grafting of OTMS to SiO_2_ surfaces to strengthen the interfacial interaction [39]. When modified SiO_2_ nanoparticles were employed, the dispersion of SiO_2_ in the PP-OTMS matrix further improved (Figure 2c–e). In Figure 2f, the *D* value increases along the alkyl chain length. These results are explained by the decrease in the cohesive attraction among SiO_2_ nanoparticles and improved compatibility with the matrix due to organic modification, and/or the prevention of re-agglomeration due to more efficient grafting of PP-OTMS (described below).

**Figure 3:**
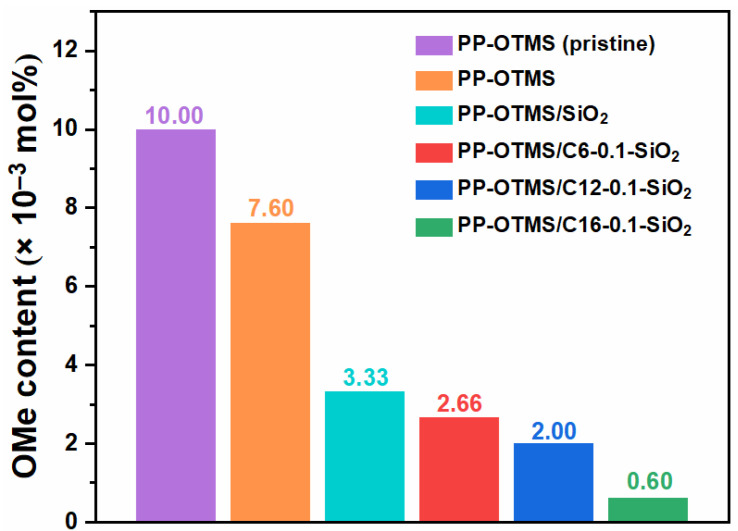
The OMe content analyzed by ^1^H NMR before and after melt mixing.

During the melt mixing process, the OMe groups of PP-OTMS can react with surface silanol groups of SiO_2_ to form Si-O-Si bonds, by which PP-OTMS grafts to the SiO_2_ nanoparticles [39]. To confirm the occurrence of in situ grafting, the OMe content before and after melt mixing was analyzed by ^1^H NMR (Figure S3). As shown in Figure 3, even in the absence of SiO_2_, a part of OMe groups was consumed during melt mixing. This suggests that the OMe groups belonging to different chains can react with each other, most plausibly via hydrolysis/condensation with the aid of residual water. In the presence of SiO_2_, the decrease in the OMe content became more pronounced, which indicated the occurrence of in situ grafting. The consumption of the OMe group was greater for modified SiO_2_ as compared to unmodified one, even though surface silanol groups were partially consumed by the organic modification. This result evidenced the importance of initial dispersion on the efficiency of the in situ grafting. The consumption of the OMe groups increased along with the alkyl chain length, in agreement with the improved dispersion parameter in Figure 2f. It is plausible that the improved dispersion of modified SiO_2_ provided more contact interfaces to promote the grafting reaction.

The influences of SiO_2_ and its surface modification on the melting and crystallization behaviors of nanocomposites were investigated by DSC. The acquired parameters, such as the melting temperature (*T*_m_), crystallization temperature (*T*_c_), and crystallinity (*X*_c_) of the nanocomposites, are summarized in Table 2. The DSC profiles during heating and cooling are respectively provided in Figures S4 and S5. In Table 2, *T*_m_ and *X*_c_ of PP-OTMS were slightly higher than those of HomoPP due to slightly higher isotacity of PP-OTMS (98 mol% *mmmm* for PP-OTMS vs. 95 mol% for HomoPP). On the other hand, *T*_c_ and *t*_1/2_^−1^ were obviously higher for PP-OTMS. This is due to the nucleation ability of PP-OTMS, which forms a long-chain branched (LCB) structure by interchain reaction during melt mixing and shows a nucleation ability similar to LCBPP [39]. The introduction of SiO_2_ to HomoPP did not cause any significant change in the thermal properties and the crystallinity of the resultant nanocomposite. Contrary, the introduction of SiO_2_ to the PP-OTMS matrix increased *T*_c_ by 3 °C and doubled the crystallization rate. The significant crystallization acceleration has been reported for the other grafted-type nanocomposites, where grafted PP chains with lower mobility facilitate nucleation [54,55]. The crystallization acceleration was also observed with modified SiO_2_, and became more pronounced for a longer alkyl chain. These results are in perfect agreement with the TEM and NMR results that the surface modification improved the dispersion of SiO_2_ nanoparticles and made the grafting reaction more efficient, in particular for a longer alkyl chain.

Tensile properties of nanocomposites were acquired using a uniaxial tensile tester. The representative stress–strain curves of the nanocomposites are provided in Figure S6. From Table 3 and Figure S7, the yield strength and elongation at break of PP-OTMS were higher than HomoPP as a consequence of LCBPP formation [56]. The addition of SiO_2_ to HomoPP did not cause the improvement in the mechanical properties, but rather deteriorated the elongation at break due to the nanoparticle agglomeration and poorly connected interfaces [38,39]. On the other hand, the introduction of SiO_2_ to PP-OTMS improved both of the Young’s modulus and yield strength as compared to PP-OTMS. Such the improvement was associated with the improved dispersion and the successful grafting of PP-OTMS onto SiO_2_ surfaces. The grafting not only improves the dispersion of SiO_2_ in PP, but also improves the interfacial bonding (hence the yield strength). Grafted chains co-crystallize with the matrix, and SiO_2_ acts as a physical crosslinker, which disappears above the melting point, so nanocomposites are melt processible [38]. This is the largest advantage of graft-type nanocomposites with identical matrix and grafted chains. In contrast, the physical crosslink structure with SiO_2_ as the core is much less flexible to deformation, which leads to a large decrease in elongation at break. This is a common disadvantage of the graft-type nanocomposites [38,39,40], and the significant decrease in the elongation at break and the toughness was also observed in PP-OTMS/SiO_2_. By exploiting modified SiO_2_ (C*x*-0.1-SiO_2_), the elongation at break was partially recovered in addition to further improvement in the yield strength. The degree of the recovery/improvement was found to be greater for a longer alkyl chain (Table 3). In general, the yield strength is largely determined by the dispersion and interfacial interactions between the polymer matrix and fillers. Hence, the higher yield strength for a longer alkyl chain reasonably arose from better SiO_2_ dispersion as well as from a larger extent of in situ grafting. Meanwhile, the elongation at break depends on the incidental presence of agglomerates or defects and the ductility of the original materials. In this light, a long alkyl chain not only provided homogeneous dispersion, but also behaved as a plasticizer to enhance the ductility of the materials, leading to the recovery of the elongation at break [26,28]. With the improvement in the yield strength and the recovery of the elongation at break, the toughness of the resultant nanocomposites was greatly enhanced along with the alkyl chain length (Table 3). The influence of the silane grafting density on mechanical properties of nanocomposites was also studied for C16 (Table 3). It was found that an excessive addition of the silane coupling agent rather deteriorated the mechanical properties. This was likely associated with the formation of a soft organic layer, which hampered the stress transfer to hard nanoparticles.

## 4. Conclusions

In this study, organically modified SiO_2_ was exploited for the fabrication of graft-type polymer nanocomposites using polypropylene having reactive functional groups (PP-OTMS) as a matrix. It was shown that the surface modification of SiO_2_ with silane coupling agents helped the dispersion of SiO_2_ to promote the in situ grafting during melt compounding of the reactive matrix with nanofillers. The influences of the alkyl chain length of silane coupling agents and the grafting amount on the properties of resultant nanocomposites were also studied. It was found that the dispersion improved with the increase in the alkyl chain length due to the improved hydrophobicity of SiO_2_ surfaces. This offered a more opportunity for the reactive groups at the PP side chain to react with the remaining surface silanol groups during melt mixing and thus to in situ graft onto nanofiller surfaces. This grafting reaction was confirmed by the significant reduction in the amount of the reactive functional groups and the observed crystallization acceleration in the resultant nanocomposites. The advantage of the combination of surface modification and in situ grafting was evidenced for the mechanical properties, in which the efficient grafting strengthened the interfacial interaction between the matrix and SiO_2_ to improve the reinforcement. Furthermore, the modification with plasticizing alkyl chains also helped to recover the toughness deterioration caused by the grafting itself. This provided the opportunity to balance the reinforcement and the toughness of the materials, which is difficult to achieve by in situ grafting alone.

## Figures and Tables

**Table 1:** Silane grafting amounts derived from TGA.

Sample	*m*(%) ^a^	Silane Grafting Density (nm^−2^) ^b^
C6-0.1-SiO_2_	1.57	1.43
C12-0.1-SiO_2_	0.89	0.81
C16-0.1-SiO_2_	0.72	0.66
C16-0.01-SiO_2_	0.13	0.12
C16-1-SiO_2_	1.03	0.94

^a^ Calculated based on Equation (3); ^b^ The surface area of SiO_2_ was measured as 110 m^2^ g^−1^.

**Table 2:** Melting and crystallization behaviors of nanocomposites analyzed by DSC.

Sample	*T*_m_ (°C)	*X*_c_ (%)	*T*_c_ (°C)	*t*_1/2_^−1 a^ (×10^−3^ s^−1^)
HomoPP	161	49	117	0.13
HomoPP/SiO_2_	161	48	118	0.21
PP-OTMS	163	53	126	2.11
PP-OTMS/SiO_2_	164	52	129	4.63
PP-OTMS/C6-0.1-SiO_2_	164	50	129	4.59
PP-OTMS/C12-0.1-SiO_2_	164	49	129	4.90
PP-OTMS/C16-0.1-SiO_2_	164	49	129	5.21

^a^ Inverse of the half time of isothermal crystallization at 144 °C.

**Table 3:** Tensile properties of nanocomposites.

Sample	Young’s Modulus (MPa)	Yield Strength (MPa)	Elongation at Break (%)	Toughness (MJ/m^3^)
HomoPP	576 ± 18	30.2 ± 0.3	24.3 ± 2.7	5.4 ± 0.8
HomoPP/SiO_2_	617 ± 46	29.6 ± 0.4	13.9 ± 1.1	2.8± 0.2
PP-OTMS	621 ± 30	34.8 ± 0.5	32.0 ± 6.5	7.9 ± 0.9
PP-OTMS/SiO_2_	639 ± 39	36.5 ± 1.5	12.2 ± 0.2	2.9 ± 0.2
PP-OTMS/C6-0.1-SiO_2_	646 ± 40	36.8 ± 0.6	14.4 ± 1.6	3.1 ± 0.6
PP-OTMS/C12-0.1-SiO_2_	657 ± 28	37.6 ± 1.1	18.1 ± 4.1	4.3 ± 1.2
PP-OTMS/C16-0.1-SiO_2_	662 ± 52	37.8 ± 1.0	20.1 ± 2.2	5.8 ± 0.7
PP-OTMS/C16-0.01-SiO_2_	633 ± 56	36.7 ± 0.8	13.8 ± 0.7	3.5 ± 0.2
PP-OTMS/C16-1-SiO_2_	620 ± 46	35.8 ± 2.8	12.9 ± 3.9	3.1 ± 1.2

## Data Availability

The data presented in this study are available on request from the corresponding author.

## Supplementary Materials

The following supporting information can be downloaded at: https://www.mdpi.com/article/10.3390/polym14030563/s1, Figure S1. TEM images of neat and surface modified SiO_2_ nanoparticles. Figure S2. ^1^H NMR spectra of HomoPP and PP-OTMS. Figure S3. ^1^H NMR of PP-OTMS and its nanocomposites before and after melt mixing. Figure S4. DSC profiles during heating. Figure S5. DSC profiles during cooling. Figure S6. Stress–strain curves. Figure S7. Tensile properties: (a) Young’s modulus, (b) yield strength, (c) elongation at break, and (d) toughness of nanocomposites.

## References

[1] Paul D.R., Robeson L.M. (2008). Polymer nanotechnology: Nanocomposites. Polymer.

[2] Gao F. (2004). Clay/polymer composites: The story. Mater. Today.

[3] Liu H., Gu S., Cao H., Li X., Jiang X., Li Y. (2019). Modification of MWNTs by the combination of Li-TFSI and MAPP: Novel strategy to high performance PP/MWNTs nanocomposites. Compos. Part B Eng..

[4] Ma P.-C., Siddiqui N.A., Marom G., Kim J.-K. (2010). Dispersion and functionalization of carbon nanotubes for polymer-based nanocomposites: A review. Compos. Part A Appl. Sci. Manuf..

[5] Palza H., Vera J., Wilhelm M., Zapata P. (2011). Spherulite growth rate in polypropylene/silica nanoparticle composites: Effect of particle morphology and compatibilizer. Macromol. Mater. Eng..

[6] Mao H., He B., Guo W., Hua L., Yang Q. (2018). Effects of nano-CaCO_3_ content on the crystallization, mechanical properties, and cell structure of PP nanocomposites in microcellular injection molding. Polymers.

[7] Stanciu N.-V., Stan F., Sandu I.-L., Fetecau C., Turcanu A.-M. (2021). Thermal, rheological, mechanical, and electrical properties of polypropylene/multi-walled carbon nanotube nanocomposites. Polymers.

[8] Navas I.O., Kamkar M., Arjmand M., Sundararaj U. (2021). Morphology evolution, molecular simulation, electrical properties, and rheology of carbon nanotube/polypropylene/polystyrene blend nanocomposites: Effect of molecular interaction between styrene-butadiene block copolymer and carbon nanotube. Polymers.

[9] Castro-Landinez J.F., Salcedo-Galan F., Medina-Perilla J.A. (2021). Polypropylene/ethylene-and polar-monomer-based copolymers/montmorillonite nanocomposites: Morphology, mechanical properties, and oxygen permeability. Polymers.

[10] Norrrahim M.N.F., Ariffin H., Arisyah T., Yasim-Anuar T.A.T., Hassan M.A., Ibrahim N.A., Yunus W.M.Z.W., Nishida H. (2021). Performance evaluation of cellulose nanofiber with residual hemicellulose as a nanofiller in polypropylene-based nanocomposite. Polymers.

[11] Velásquez E., Espinoza S., Valenzuela X., Garrido L., Galotto M.J., Guarda A., Dicastillo C.L.D. (2021). Effect of organic modifier types on the physical-mechanical properties and overall migration of post-consumer polypropylene/clay nanocomposites for food packaging. Polymers.

[12] Constant-Mandiola B., Aguilar-Bolados H., Geshev J., Quíjada R. (2021). Study of the influence of magnetite nanoparticles supported on thermally reduced graphene oxide as filler on the mechanical and magnetic properties of polypropylene and polylactic acid nanocomposites. Polymers.

[13] Vidakis N., Petousis M., Velidakis E., Tzounis L., Mountakis N., Korlos A., Fischer-Griffiths P.E., Grammatikos S. (2021). On the mechanical response of silicon dioxide nanofiller concentration on fused filament fabrication 3D printed isotactic polypropylene nanocomposites. Polymers.

[14] Titone V., Mistretta M.C., Botta L., Mantia F.P.L. (2021). Investigation on the properties and on the photo-oxidation behaviour of polypropylene/fumed silica nanocomposites. Polymers.

[15] Jung B.-N., Jung H.-W., Kang D.-H., Kim G.-H., Shim J.-K. (2021). A study on the oxygen permeability behavior of nanoclay in a polypropylene/nanoclay nanocomposite by biaxial stretching. Polymers.

[16] Naseem S., Wießner S., Kühnert I., Leuteritz A. (2021). Layered double hydroxide (MgFeAl-LDH)-based polypropylene (PP) nanocomposite: Mechanical properties and thermal degradation. Polymers.

[17] Bikiaris D.N., Vassiliou A., Pavlidou E., Karayannidis G.P. (2005). Compatibilisation effect of PP-g-MA copolymer on iPP/SiO_2_ nanocomposites prepared by melt mixing. Eur. Polym. J..

[18] Li W., Karger-Kocsis J., Thomann R. (2009). Compatibilization effect of TiO_2_ nanoparticles on the phase structure of PET/PP/TiO_2_ nanocomposites. J. Polym. Sci. Part B Polym. Phys..

[19] Chiu F.-C., Yen H.-Z., Lee C.-E. (2010). Characterization of PP/HDPE blend-based nanocomposites using different maleated polyolefins as compatibilizers. Polym. Test..

[20] Liborio P., Oliveira V.A., Maria de Fatima V.M. (2015). New chemical treatment of bentonite for the preparation of polypropylene nanocomposites by melt intercalation. Appl. Clay Sci..

[21] Ataeefard M., Moradian S. (2011). Surface properties of polypropylene/organoclay nanocomposites. Appl. Surf. Sci..

[22] Peng P., Yang Z., Wu M., Zhang Q., Chen G. (2013). Effect of montmorillonoite modification and maleic anhydride-grafted polypropylene on the microstructure and mechanical properties of polypropylene/montmorillonoite nanocomposites. J. Appl. Polym. Sci..

[23] Maiti P., Nam P.H., Okamoto M. (2002). Influence of crystallization on intercalation, morphology, and mechanical properties of polypropylene/clay nanocomposites. Macromolecules.

[24] Al-Saleh M.A., Yussuf A.A., Al-Enezi S., Kazemi R., Wahit M.U., Al-Shammari T., Al-Banna A. (2019). Polypropylene/graphene nanocomposites: Effects of GNP loading and compatibilizers on the mechanical and thermal properties. Materials.

[25] Ataeefard M., Moradian S. (2011). Polypropylene/organoclay nanocomposites: Effects of clay content on properties. Polym. Plast. Technol. Eng..

[26] Ahn S.H., Kim S.H., Lee S.G. (2004). Surface-modified silica nanoparticle-reinforced poly(ethylene 2,6-naphthalate). J. Appl. Polym. Sci..

[27] Lin O.H., Akil H.M., Mohd Ishak Z.A. (2011). Surface-activated nanosilica treated with silane coupling agents/polypropylene composites: Mechanical, morphological, and thermal studies. Polym. Compos..

[28] Zhou R.-J., Burkhart T. (2010). Polypropylene/SiO_2_ nanocomposites filled with different nanosilicas: Thermal and mechanical properties, morphology and interphase characterization. J. Mater. Sci..

[29] Rungta A., Natarajan B., Neely T., Dukes D., Schadler L.S., Benicewicz B.C. (2012). Grafting bimodal polymer brushes on nanoparticles using controlled radical polymerization. Macromolecules.

[30] Li C., Benicewicz B.C. (2005). Synthesis of well-defined polymer brushes grafted onto silica nanoparticles via surface reversible addition-fragmentation chain transfer polymerization. Macromolecules.

[31] Ranjan R., Brittain W.J. (2008). Synthesis of high density polymer brushes on nanoparticles by combined RAFT polymerization and click chemistry. Macromol. Rapid Commun..

[32] Wang H., Fu Z., Zhao X., Li Y., Li J. (2017). Reactive nanoparticles compatibilized immiscible polymer blends: Synthesis of reactive SiO_2_ with long poly(methyl methacrylate) chains and the in situ formation of janus SiO_2_ nanoparticles anchored exclusively at the interface. ACS Appl. Mater. Interfaces.

[33] Wang H., Yang X., Fu Z., Zhao X., Li Y., Li J. (2017). Rheology of nanosilica-compatibilized immiscible polymer blends: Formation of a “heterogeneous network” facilitated by interfacially anchored hybrid nanosilica. Macromolecules.

[34] Milani M.A., González D., Quijada R., Basso N.R.S., Cerrada M.L., Azambuja D.S., Galland G.B. (2013). Polypopylene/graphene nanosheet nanocomposites by in situ polymerization: Synthesis, characterization and fundamental properties. Compos. Sci. Technol..

[35] Zhang X., Wada T., Chammingkwan P., Thakur A., Taniike T. (2019). Cooperative influences of nanoparticle localization and phase coarsening on thermal conductivity of polypropylene/polyolefin elastomer blends. Compos. Part A Appl. Sci. Manuf..

[36] Zhang X., Xia X., You H., Wada T., Chammingkwan P., Thakur A., Taniike T. (2020). Design of continuous segregated polypropylene/Al_2_O_3_ nanocomposites and impact of controlled Al_2_O_3_ distribution on thermal conductivity. Compos. Part A Appl. Sci. Manuf..

[37] Zhang X., Maira B., Hashimoto Y., Wada T., Chammingkwan P., Thakur A., Taniike T. (2019). Selective localization of aluminum oxide at interface and its effect on thermal conductivity in polypropylene/polyolefin elastomer blends. Compos. Part B Eng..

[38] Taniike T., Toyonaga M., Terano M. (2014). Polypropylene-grafted nanoparticles as a promising strategy for boosting physical properties of polypropylene-based nanocomposites. Polymer.

[39] Kurahashi E., Wada T., Nagai T., Chammingkwan P., Terano M., Taniike T. (2018). Synthesis of polypropylene functionalized with a trace amount of reactive functional groups and its utilization in graft-type nanocomposites. Polymer.

[40] Zhang G., Li H., Antensteiner M., Mike Chung T.C. (2015). Synthesis of functional polypropylene containing hindered phenol stabilizers and applications in metallized polymer film capacitors. Macromolecules.

[41] Jing Y., Niu H., Li Y. (2019). Improved ethylene-propylene rubber/silica interface via in-situ polymerization. Polymer.

[42] Toyonaga M., Chammingkwan P., Terano M., Taniike T. (2016). Well-defined polypropylene/polypropylene-grafted silica nanocomposites: Roles of number and molecular weight of grafted chains on mechanistic reinforcement. Polymers.

[43] Mehdiabadi S., Soares J.B.P. (2011). Production of ethylene/α-Olefin/1,9-decadiene copolymers with complex microstructures using a two-stage polymerization process. Macromolecules.

[44] Guo Q., Zhu P., Li G., Lu D., Sun R., Wong C.-P. (2016). Effects of surface-modified alkyl chain length of silica fillers on the rheological and thermal mechanical properties of underfill. IEEE Trans. Compon. Packag Manuf. Technol..

[45] Sriboonruang A., Kumpika T., Kantarak E., Sroila W., Singjai P., Lawan N., Muangpile S., Thongsuwan W. (2019). Isomer effect on chemical reactivity and superhydrophobicity of chlorosilane modified SiO_2_ nanoparticles prepared by one-step reaction. Mater. Lett..

[46] Glaskova T., Zarrelli M., Borisova A., Timchenko K., Aniskevich A., Giordano M. (2011). Method of quantitative analysis of filler dispersion in composite systems with spherical inclusions. Compos. Sci. Technol..

[47] Ogihara H., Xie J., Saji T. (2013). Factors determining wettability of superhydrophobic paper prepared by spraying nanoparticle suspensions. Colloids Surf. A.

[48] Yang X., Zhu L., Chen Y., Bao B., Xu J., Zhou W. (2016). Controlled hydrophilic/hydrophobic property of silica films by manipulating the hydrolysis and condensation of tetraethoxysilane. Appl. Surf. Sci..

[49] Wang Y., Zhang L., Hu Y., Li C. (2015). In situ surface functionalization of hydrophilic silica nanoparticles via flame spray process. J. Mater. Sci. Technol..

[50] Yuan Y., Duan Y., Zuo Z., Yang L., Liao R. (2017). Novel, stable and durable superhydrophobic film on glass prepared by RF magnetron sputtering. Mater. Lett..

[51] Mueller R., Kammler H.K., Wegner K., Pratsinis S.E. (2003). OH surface density of SiO_2_ and TiO_2_ by thermogravimetric analysis. Langmuir.

[52] Ji T., Ma C., Brisbin L., Mu L., Robertson C.G., Dong Y., Zhu J. (2017). Organosilane grafted silica: Quantitative correlation of microscopic surface characters and macroscopic surface properties. Appl. Surf. Sci..

[53] Chen W., Karde V., Cheng T.N.H., Ramli S.S., Heng J.Y.Y. (2020). Surface hydrophobicity: Effect of alkyl chain length and network homogeneity. Front. Chem. Sci. Eng..

[54] Tabatabaei S.H., Carreau P.J., Ajji A. (2009). Rheological and thermal properties of blends of a long-chain branched polypropylene and different linear polypropylenes. Chem. Eng. Sci..

[55] Tian J., Yu W., Zhou C. (2007). Crystallization behaviors of linear and long chain branched polypropylene. J. Appl. Polym. Sci..

[56] Zhao W., Huang Y., Liao X., Yang Q. (2013). The molecular structure characteristics of long chain branched polypropylene and its effects on non-isothermal crystallization and mechanical properties. Polymer.

