# Supplementary material for: Enhancing Mechanical Properties of Graft-Type Nanocomposites Using Organically Modified SiO2 and Polypropylene Containing Reactive Methoxy Groups
Source: Polymers (Basel). 2022 Jan 30;14(3):563. doi: 10.3390/polym14030563 (PMC8838797; doi:10.3390/polym14030563)
Supplement: Supplementary file 1 [file polymers-14-00563-s001.zip › polymers-1565339-supplementary.pdf]

Supplementary Information

Table of contents

Figure S1. ^{1}H NMR spectra of HomoPP and PP-OTMS.

Figure S2. TEM images of neat and surface modified SiO_2 nanoparticles.

Figure S3. ^{1}H NMR of PP-OTMS and its nanocomposites before and after melt mixing.

Figure S4. DSC profiles during heating.

Figure S5. DSC profiles during cooling.

Figure S6. Stress-strain curves.

Figure S7. Tensile properties: (a) Young's modulus, (b) yield strength, (c) elongation at break, and (d) toughness of nanocomposites.

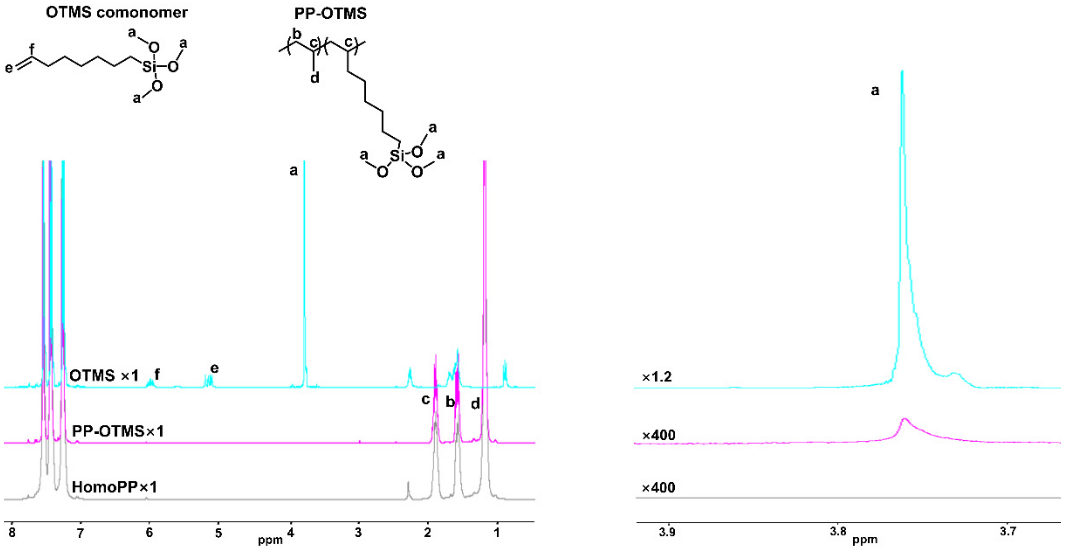

Figure S1. ^{1}H NMR spectra of HomoPP and PP-OTMS. The right figure enlarges the methoxy region.

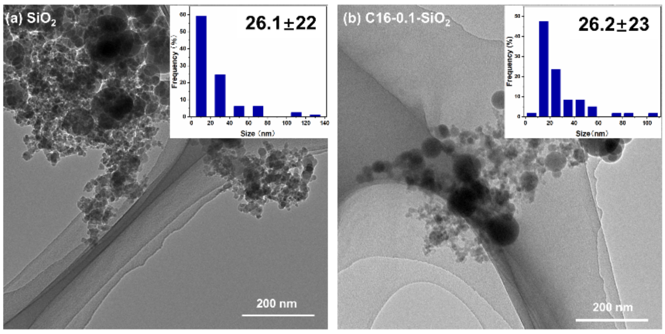

Figure S2. TEM images of neat and surface modified SiO_2 nanoparticles.

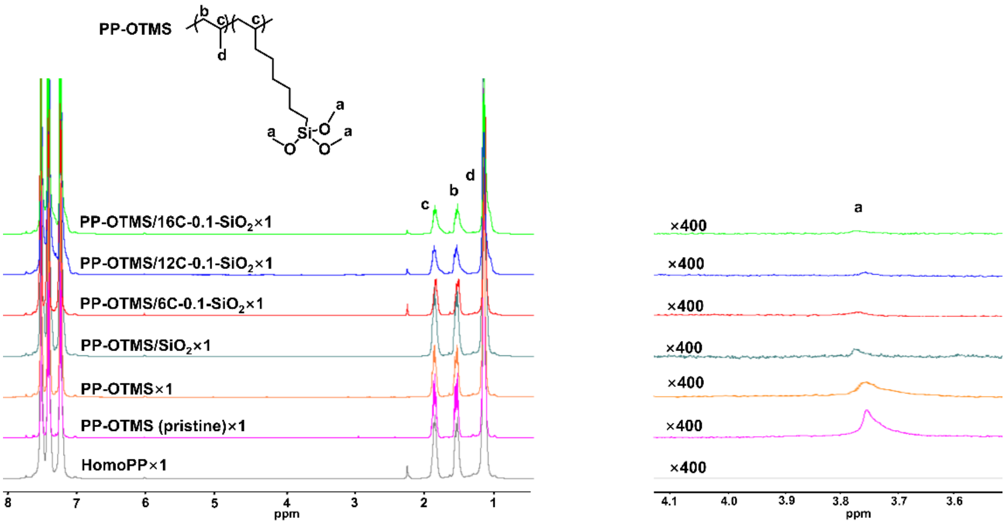

Figure S3. ^{1}H NMR of PP-OTMS and its nanocomposites before and after melt mixing. The right figure enlarges the methoxy region.

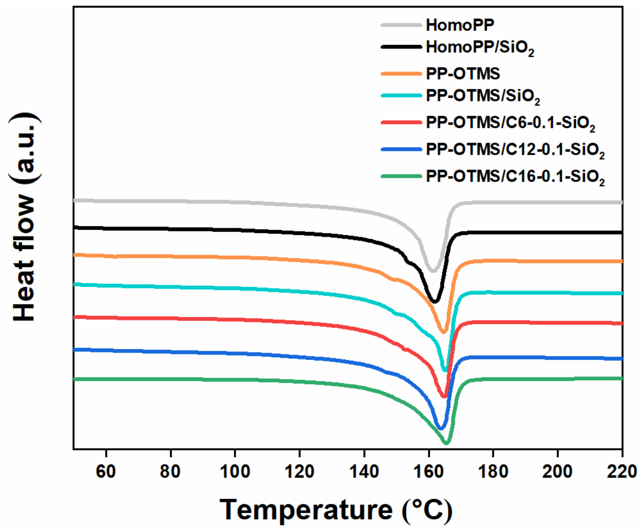

Figure S4. DSC profiles during heating.

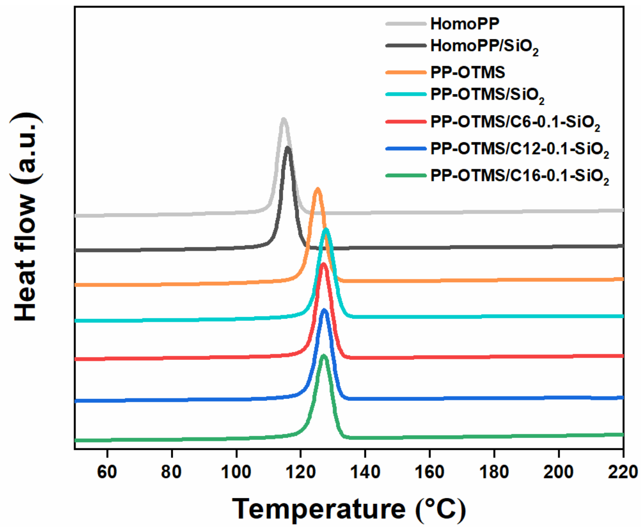

Figure S5. DSC profiles during cooling. Profiles are vertically shifted for visual clarity.

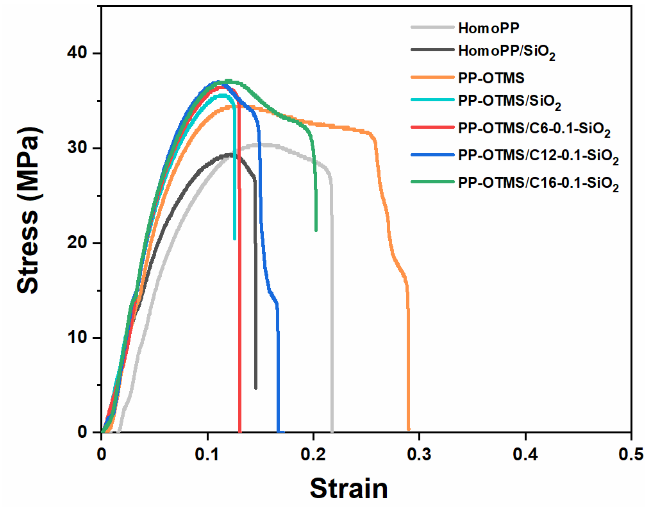

Figure S6. Stress-strain curves.

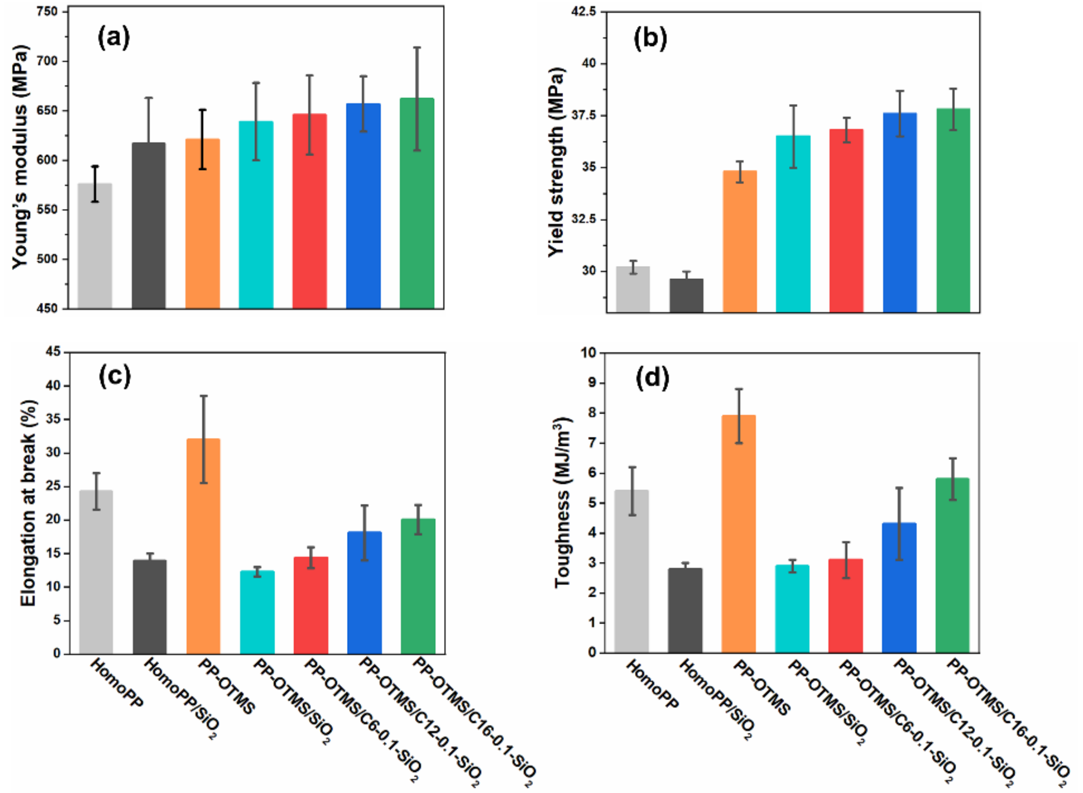

Figure S7. Tensile properties: (**a**) Young's modulus, (**b**) yield strength, (**c**) elongation at break, and (**d**) toughness of nanocomposites.
